# MicroRNAs provide negative feedback and stability in gene regulatory network models of cell-state transitions

**DOI:** 10.3389/freae.2024.1473789

**Published:** 2024-10-02

**Authors:** Milad Razavi-Mohseni, Michael A. Beer

**Affiliations:** Department of Biomedical Engineering and McKusick-Nathans Department of Genetic Medicine, Johns Hopkins University, Baltimore, MD, United States

**Keywords:** gene regulatory network (GRN), microRNA, machine learning, cell-state transitions, dynamic network modeling, enhancers

## Abstract

The development of multicellular organisms occurs through a series of cell state transitions controlled by gene regulatory networks. Central to these networks are transcription factors (TFs) which bind enhancers and activate the expression of other genes, some of which are also TFs. Gene regulatory networks (GRN) connect TFs and enhancers in a nonlinear circuit capable of producing complex behavior such as bifurcations between stable cell states. Our dynamic network modelling of the Embryonic Stem Cell (ESC) to Definitive Endoderm (DE) transition requires an as yet unknown negative feedback mechanism for stability. Here, we show that cell state specific microRNAs (miRNAs) can provide this negative feedback by inactivating other cell lineage determining TFs (ESC or DE) during the transition. Our model provides a mechanism to maintain stable cell states without requiring a large set of cell-type-specific repressive TFs, of which there are fewer known examples than activators. In support of this model, we use computational models and analyze gene and miRNA expression and chromatin accessibility data from human cell lines to detect enhancers activating the miRNAs consistent with our network model. Our analysis highlights the interplay between TFs and miRNAs during ESC to DE transition and proposes a novel model for gene regulation.

## Introduction

Cell state transitions and their proper regulation are central to the development of multicellular organisms. These transitions are controlled by gene regulatory networks which exhibit complex behavior in response to developmental and environmental perturbations via the nonlinear interactions between transcription factor (TF) binding at enhancers that activate other TF genes. We refer to lineage-specific TFs as core TFs, and their transcription factor binding sites (TFBS) can quantitatively predict the active enhancers (accessible peaks) in that cell type. Differentiated cell types are stable states of these gene regulatory networks (Davidson, 2010; Beer et al., 2020). In addition to being important for understanding congenital developmental disorders, gene regulatory networks are disrupted in cancer (Xing et al., 2020; Xu et al., 2023; Sheng et al., 2021; Ho et al., 2023; Razavi-Mohseni et al., 2024), and quantitative models may aid the identification of effective therapeutic targets.

We have recently developed quantitative dynamic gene regulatory network (GRN) models (Luo et al., 2023) of this cell state transition process, which reproduce many intriguing experimental observations in the embryonic stem cell (ESC) to definitive endoderm (DE) transition (Luo et al., 2023). In these experiments, the differentiation from ESC to DE is induced while perturbing enhancers with CRISPRi. Perturbation of single enhancers flanking core DE TF genes caused a significant decrease in expression of the core DE TF genes, but the transcriptional response was transient, and eventually fully recovered to unperturbed levels, in spite of the fact that ATAC-seq verified that the CRISPRi targeted enhancers remained inaccessible (Luo et al., 2023). This dynamic effect is explained in a nonlinear model by autoregulation of the cell-specific TFs (of which there are many, perhaps 5-10 per cell type) and redundant enhancers (of which there are also typically 5-10 enhancers per core TF gene). If only one enhancer is perturbed, the other enhancers are usually sufficient to activate the core TFs. In contrast, if the promoter is perturbed, the gene remains inactive. An additional component of this modeling is that all enhancers within a CTCF loop, or topologically associating domain (TAD), can activate the target gene, while those outside a CTCF loop have little or no effect (Luo et al., 2023). This observation greatly simplifies the complex problem of enhancer-promoter interactions (Xi and Beer, 2018; Gschwind et al., 2023; Yao et al., 2024) needed for modeling of gene regulatory networks, as CTCF binding and its action as anchors to cohesin extruded loops are, to a large degree, predictable with simple models (Xi and Beer, 2021) and are largely independent of cell type.

However, in this model (Luo et al., 2023) we only focused on the activation of core DE TF genes, and not the concomitant inactivation of the core ESC TF genes. In our earlier modeling (Li et al., 2019; Beer et al., 2020), we were able to model stable bifurcations between cell states by including negative feedback between core DE TFs (GATA6, SOX17, EOMES, MIXL1) and core ESC TFs (POU5F1(OCT4), SOX2, NANOG), as shown in Figure 1A (TF expression shown in Supplementary Figure S1). However, this mechanism requires a set of repressive TFs, or a repressive configuration of otherwise activating TFs, specific to each individual cell type, which may be difficult to satisfy given the relatively few known repressive TFs compared to activators. Our large-scale machine learning analysis of chromatin accessibility in a broad range of cell types and tissues in the ENCODE project identifies TFBS for some known repressive TFs, but we find that the majority of cell-specific TFBS signals we learn are activating. To be clear, by this we mean that the predictive cell-specific sequence features (TFBS) are dominated by those that have a positive model weight, increase the accessibility of peaks, and are recognized as TFBS active in the cell type. Yet it is experimentally clear that inducing the DE state somehow inactivates the ESC TFs. We do not know the direct consequences of having both ESC and DE TFs active at the same time, but it likely disrupts the proper expression of downstream genes required for cellular viability and function. Similarly, prior to the induction of DE, without a repressive effect of ESC TFs on DE TFs, autoregulation of DE TFs could inappropriately activate in response to signaling fluctuations or noise. Thus, our modeling implies that stable and precise development of all cell types (not only ESC and DE) requires some negative feedback acting on core TFs of cell types immediately upstream and downstream in the lineage.

**FIGURE 1:**
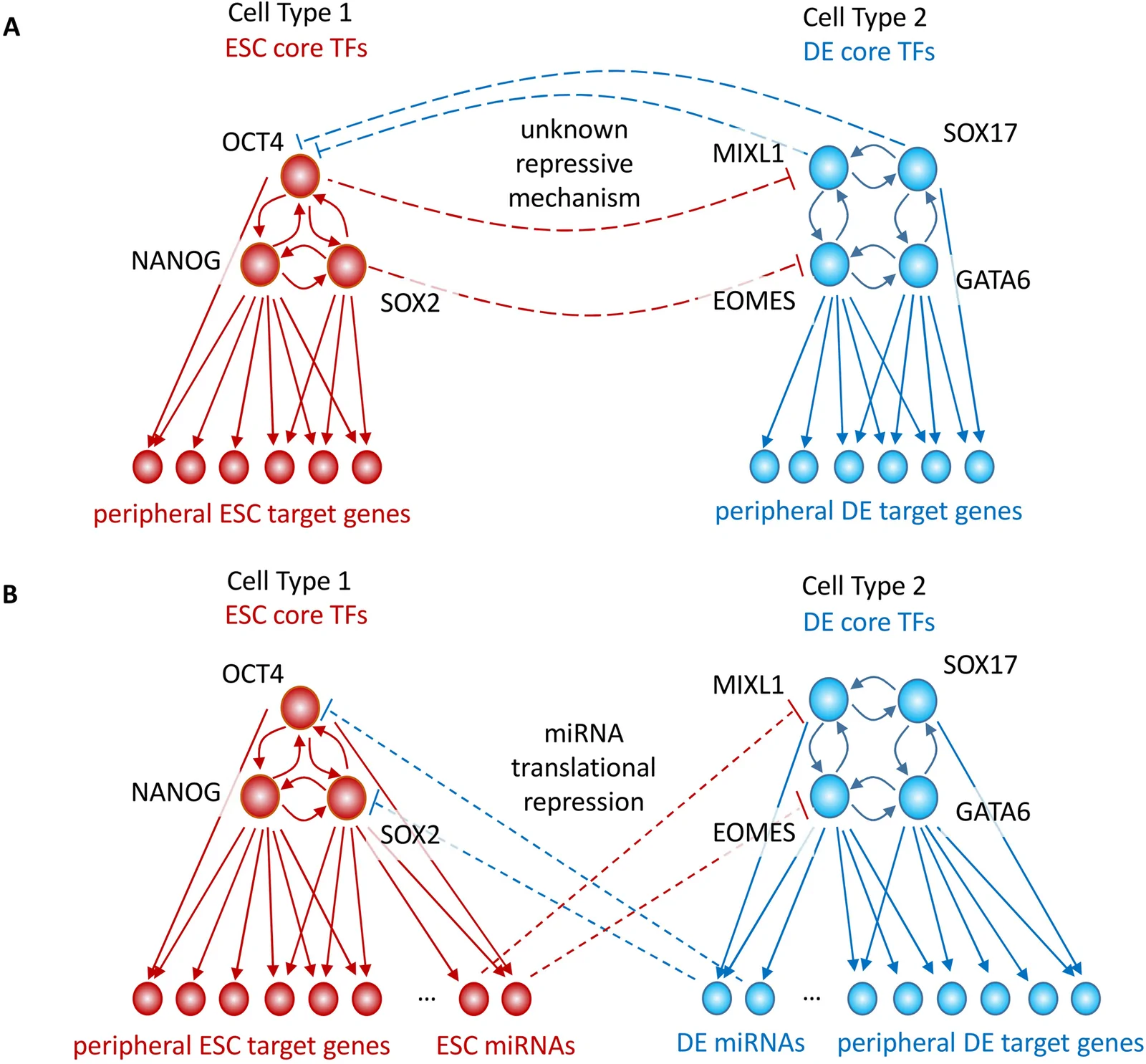
GRN models of cell-state transitions occur via activation of cell-specific TFs, but also require repressive mechanisms to inactivate TFs of the previous cell-state. **(A)** Autoregulatory GRN cell state transitions require an unknown negative feedback mechanism of cell type 1’s TFs on cell type 2’s TFs, and *vice versa*. **(B)** miRNAs activated in one cell type can provide this negative feedback mechanism by targeting the 3′ UTRs and translationally repressing the TFs of the other cell type; Gene regulatory network (GRN), microRNA (miRNA), embryonic stem cell (ESC), definitive endoderm (DE).

While many TF genes regulate gene expression through activation, other classes of genes, such as microRNAs (miRNAs), affect gene regulation largely through direct translational repression of their targets. miRNAs are ∼22 nucleotide-long non-coding RNAs which, through their seed regions (∼7 nucleotides), bind to the 3′ untranslated region (3′ UTR) of their target mRNA and reduce the rate of translation (Lee et al., 1993; Bartel, 2004; Lewis et al., 2005). miRNAs play important roles in various biological processes such as apoptosis, cancer progression and embryonic differentiation (Chang et al., 2007; Hayes et al., 2014; Tay et al., 2008).

In this paper, we will present analysis in support of the hypothesis that miRNAs can act as a required negative feedback mechanism to prevent the misexpression of TFs whose activation is associated with immediate precursor and/or downstream cell types in the lineage. The pervasive autoregulation of core TFs makes activation of neighboring cell types susceptible to signaling noise and fluctuations which could disrupt normal development. We will show that negative feedback is required to repress precursor and downstream cell type TFs and provide stability to cell fates. As shown schematically in Figure 1B, this could be achieved if DE TFs transcriptionally activate miRNAs which translationally repress ESC TFs, and if ESC TFs transcriptionally activate miRNAs which translationally repress DE TFs. Earlier work has shown that miRNAs can produce bifurcated cell-state transitions, but did not directly model core TF or miRNA enhancer activation (Zhao et al., 2019; Lai et al., 2016). DICER1 is required for human ESC renewal (Teijeiro et al., 2018), consistent with the possibility that lack of miRNA repression induces inappropriate expression of DE or other germ layer TFs and leads to slow cell death over time. We will present machine learning analysis of functional epigenomic datasets in the early ESC to DE transition in support of miRNAs potentially acting as the required negative feedback mechanism through enhancer-driven differential miRNA activation.

## Results

### Machine learning detects many activating TFs and few repressive TFs

We used a machine learning approach, gapped-kmer SVM (gkm-SVM) (Ghandi et al., 2014a; Beer et al., 2020; Lee et al., 2015) to identify TFBS driving variations in chromatin accessibility during cell state transitions. We chose gkm-SVM for its combination of predictive accuracy and interpretability (Ghandi et al., 2014a; Beer et al., 2020; Lee et al., 2015; Shigaki et al., 2019; Beer, 2017; Yan et al., 2021; Kreimer et al., 2017; Gate et al., 2018; Mo et al., 2016; Jain et al., 2024), and because of its advantages relative to deep neural networks (DNNs) when training on smaller numbers of differentially active peaks during a transition between related cell types (∼2000 peaks). However, we have shown that gkm-SVM and DNNs detect similar features when DNNs produce a robust model (Shigaki, 2024). We trained a gkm-SVM model on the top 10,000 distal ATAC-seq peaks against random negative genomic sequences, following our standard pipeline (Beer et al., 2020), for ESC and DE (after 2 days of differentiation, DE-D2). Each gkm-SVM model summarizes the TFBS required to classify the ATAC-seq peaks in a vector of gapped kmer weights which can predict the accessibility of the sequences. These weight vectors have a tail of large positive weight kmers which map to activating TFs expressed or active in the cell type, and a much weaker tail of negative weight or repressive TFs, as shown in Figure 2A for ESC and DE. In Figure 2A, we also show the average of all weight vectors trained from every ENCODE DNase-seq dataset (1270 cell/tissues), and again find an imbalance between activating and repressive TFBS. Within the repressive tail of negative weight kmers learned in the full ENCODE set of tissues, we do learn kmers mapping to known repressive binding sites, most commonly SNAI1/2, TWIST, and ZEB1/2 (5′-CACCT-3′ motif) which are repressors known to play a key role in EMT and cancer (Craene and Berx, 2013; Vandewalle et al., 2008); GLI3, GLIS2, and ZBTB7A (5′-GACCCC-3′ motif) known to have a repressive role in Hedgehog signaling (Lex et al., 2022; 2020) and fetal hemoglobin switching (Liu et al., 2021); and nuclear hormone receptor monomer and dimer elements (5′-AGGTCA-3′) bound by large families of co-repressors (Perissi et al., 2010). We also learn non-ZEB-like E-box motifs (5′-CAGNTG-3′) which are usually activating, but repressive in heart and fibroblasts, where they could be bound by E-box binding helix-loop-helix TFs (e.g., E47, E2A, also known as TCF3) and targeted by repressive ID factors (Perk et al., 2005). Although we detect several well documented repressive TFBS, in all of our ENCODE models the negative weight tail of repressive TFBS is only slightly enriched above the expected null normal distribution, while the positive weight tail is dramatically enriched in all cell/tissues and contains hundreds of activating cell-specific TFs (5-10 per cell/tissue). In addition, we find the repressive TFBS in broader sets of cell types and tissues than the activators, so while they are clearly playing an important repressive role, there may not be cell-specific repressors unique to each cell type. For the specific case of the ESC to DE cell-state transition, we trained models on ESC and DE ATAC-seq peaks, and extracted motifs from each weight distribution (Methods). We find motifs for the expected regulators OCT4-SOX2-NANOG in ESC and GATA in DE (Figure 2B), but we found no or very weak negative weight motifs. Consistent with the results from our machine learning models, a systematic study of TF binding in HepG2 cells found that 34 out of 35 TFs with strongly reproducible effects on expression were activating, while only REST was repressive (fraction significant >0.8, |median effect estimate|>0.5) (Moyers et al., 2023). The miRNA repression analyzed in this paper is an alternative repressive mechanism.

**FIGURE 2:**
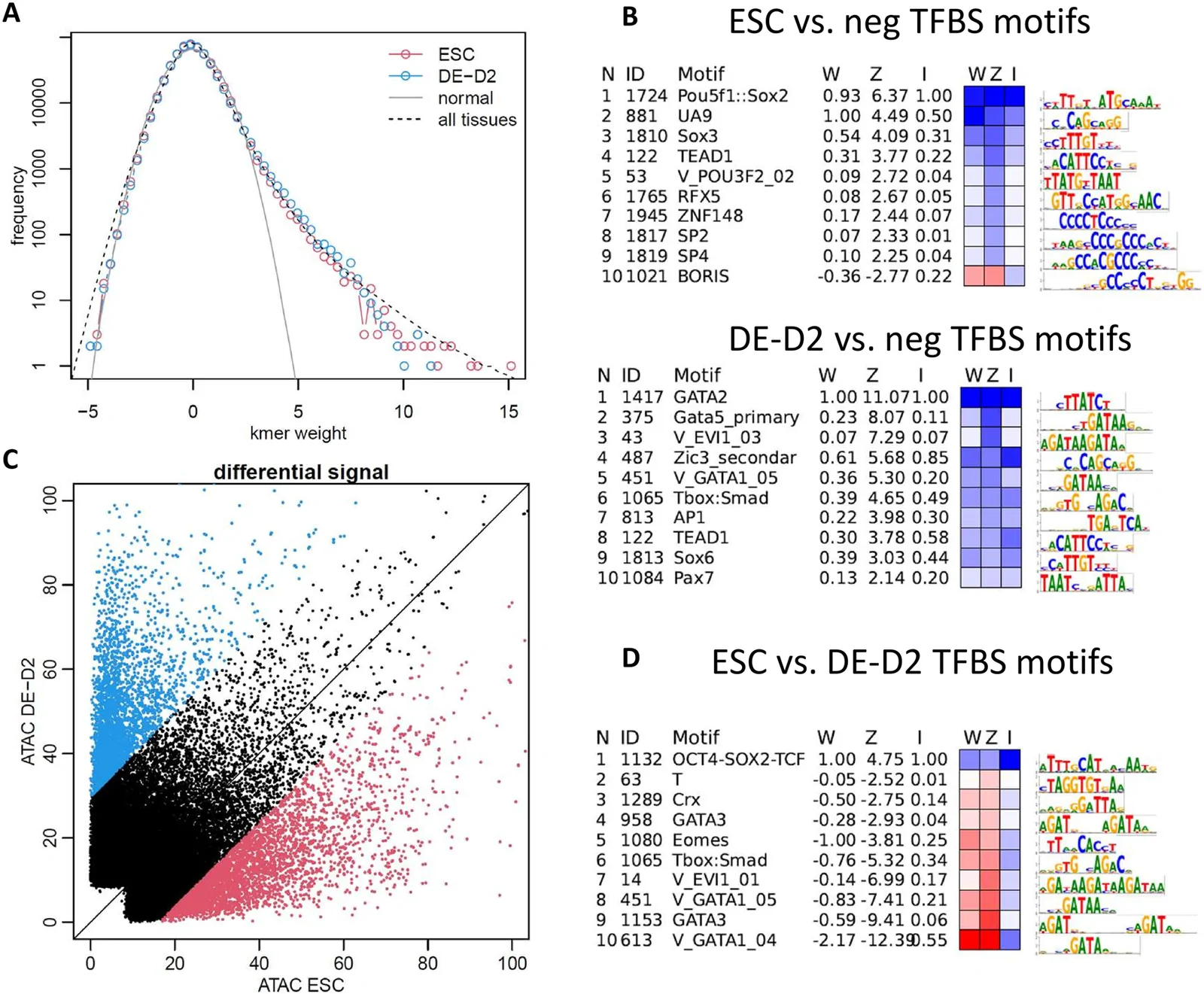
Sequence features identified by machine learning models can explain chromatin accessibility profiles during cell-state transitions with many activating TFBS, and very few repressive TFBS. **(A)** Machine learning models detect a strong tail of activating positive weight kmers mapping to TFBS in ESC, DE, and in all ENCODE cell/tissues, with only a few repressive TFBS detected (little deviation from expected null normal distribution on the negative tail). **(B)** gkm-SVM models detect expected regulators OCT4-SOX2-NANOG in ESC, and GATA in DE and only weak negative weight signals. **(C)** When trained on differentially active peaks in ESC vs. DE-D2, again only the activating signals are learned, with **(D)** OCT4-SOX2-NANOG with positive weight and GATA and EOMES with negative weight; DE day 2 (DE-D2), 10k randomly generated GC-matched genomic sequences (neg); LASSO regression weight on the gkm-SVM weight space is denoted by “W.” “Z” is the Z-score of the motif in gkm-SVM weight distribution. “I” is the relative error increase after deletion of the particular motif from the motif list explaining the weight vector.

We next trained a model on the most differentially active peaks in ESC vs. DE (n = 3000 in each set). Now OCT4-SOX2-NANOG is learned as a positive signal, and GATA, EOMES, SMAD, and MIXL1 (Crx homeodomain motif) are learned as negative signals. This training design (ESC peaks vs. DE peaks) more sensitively detects the motifs required to explain all the accessibility changes induced during the cell-state transition from ESC to DE. Again, the known activating TFBS signals OCT4-SOX2-NANOG and GATA, EOMES, SMAD, and MIXL1 are learned (the same motifs learned when trained against negative genomic regions are learned as contributing positively to that cell type’s differentially active peaks). If repressive TFs were contributing broadly to changes in ESC and DE chromatin accessibility, we would expect to see them. In some cases, we do learn an EBOX motif (5′-CANNTG-3′, which as discussed above can be repressive in some cell types) as contributing to ESC specific peaks, but this is most likely bound by MYC, which is strongly expressed in ESC but is downregulated in DE. This analysis does not preclude the possibility that DE TFs are activating a repressive TF that is only binding at a few core ESC TF enhancers (those flanking POU5F1 (OCT4), SOX2, and NANOG), but if this were a dominant mechanism, we would also expect to see the repressive TF having an effect at other peaks which have weak binding sites for the TF, and thus should appear in the weight vector. In conclusion, we see little evidence for a strong repressive TF expressed in DE which shuts down the ESC core TF enhancers, and little evidence for a strong repressive TF expressed in ESC which keeps the DE TFs repressed. The PRC2 complex may play this repressive role by being recruited to specific promoters, but how the complex achieves cell-specificity is unclear (Youmans et al., 2021).

### Dynamic gene regulatory network modeling

The gene regulatory network model (Luo et al., 2023) we developed to understand the dynamic effect of CRISPRi enhancer perturbation is a two-state transcriptional model with a large enhancer activated transcription rate, $e_{1}$ , and a low basal transcription rate, $e_{0}$ (see Figure 3A for GRN model variables). When we learn the core TFs in any cell type with gkm-SVM, we also find binding sites for multiple core TFs in each core TF enhancer. For simplicity, we model this cooperative autoregulatory activity by assuming that all core TFs are activated together, and we call this activation $\psi_{1}\left(t\right)$ for cell type 1 and $\psi_{2}\left(t\right)$ for cell type 2. $\psi \left(t\right)$ describes how the concentration of core TFs for each cell type vary as a function of time (Methods). Each cell type’s enhancers are active proportional to $\frac{c\psi^{3}}{b+c\psi^{3}}$ and inactive proportional to $\frac{b}{b+c\psi^{3}}$ . We will assume that miRNAs are similarly transcriptionally activated by enhancers, with the same rates, and repress the other cell type’s core TF activity with an additional rate d . Relaxing the assumption that core TFs and miRNAs are transcribed at the same rates $e_{1}$ and $e_{0}$ does not qualitatively change our findings. The translational repression rate, d , includes miRNA processing and nuclear transport. Together this yields two coupled nonlinear differential equations for $\psi_{1}$ and $\psi_{2}$ as shown in Figure 3A. We have also added a stimulus, $\delta \left(t\right)$ , which acts at a subset of enhancers to induce the transition from cell type 1 to 2 by activating enhancers of cell type 2 through a mechanism independent of $\psi_{2}$ . Further details of the model are provided in Methods.

**FIGURE 3:**
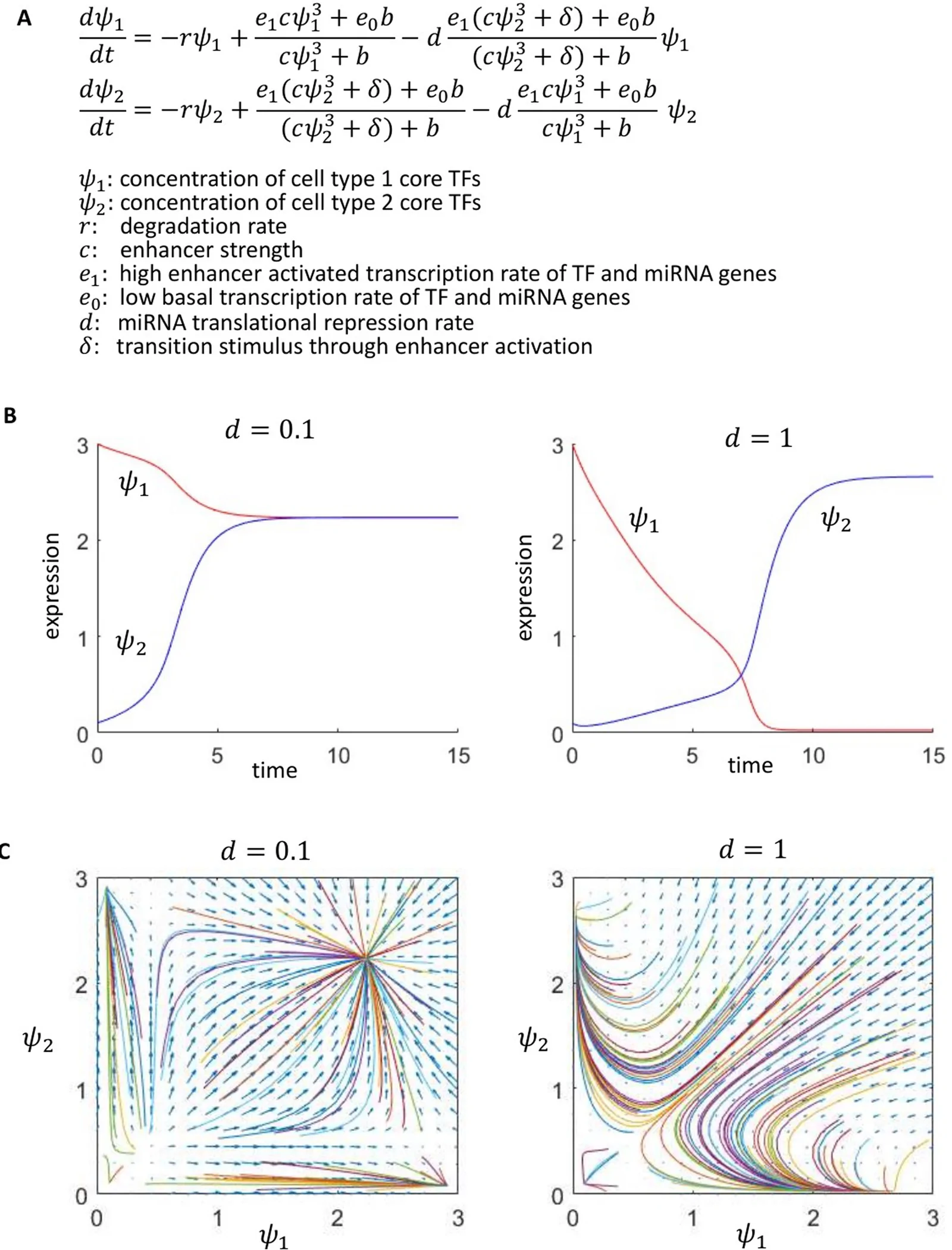
A GRN model with miRNAs activated by enhancers in one cell type, which target TFs of the other cell type, can produce stable cell-state bifurcations. **(A)** Two-state autoregulatory GRN model with miRNA negative feedback. **(B)** With weak negative feedback (*d* = 0.1), cell type 1’s TFs remain on after a stimulus, δ , activates cell type 2’s TFs. With modest negative feedback (*d* = 1), cell type 1’s TFs are repressed as cell type 2’s TFs are activated. **(C)** Phase plane analysis shows a stable fixed point with both cell types’ TFs activated at *d* = 0.1 $\left(\psi_{1},\psi_{2}\right)=($ 2.3,2.3), which becomes unstable with miRNA repression (*d* = 1).

In the limit d=0 , these two equations are completely uncoupled, and each has a stable OFF state at $\psi =e_{0}/r$ and a stable ON state at $\psi =e_{1}/r$ . When initial conditions are near the OFF state, a stimulus is required to transition into the ON state, but for d=0 , and even for small d , it is possible to activate $\psi_{1}$ and $\psi_{2}$ at the same time. As discussed in the introduction, this likely causes inappropriate expression of many downstream genes and interferes with normal cell function and viability. For small d=0.1 , with a stimulus $\delta_{0}=0.3$ (see Methods) sufficient to induce the activation of cell type 2, $\psi_{2}$ becomes activated (Figure 3B, left), but cell type 1 remains active. In contrast, for larger miRNA repression of cell type 1’s TFs d=1.0 , at the same stimulus ( $\delta_{0}=0.3$ ), when $\psi_{2}$ becomes activated, the activation of miRNAs repressing $\psi_{1}$ TFs are sufficient to drive $\psi_{1}$ fully to the OFF state (Figure 3B, right).

Further understanding of this behavior is provided by phase plane analysis in Figure 3C. For d=0.1 , there is a stable fixed point at $\left(\psi_{1},\psi_{2}\right)=($ 2.3,2.3) (Figure 3C, left), showing that a stable solution exists with both ESC and DE TFs activated. With sufficiently strong negative feedback, this stable fixed point disappears ( d>0.4 ), and the only three stable fixed points are with $\psi_{1}$ in the ON state and $\psi_{2}$ in the OFF state $\left(\psi_{1},\psi_{2}\right)=($ 3,0), $\psi_{1}$ in the OFF state and $\psi_{2}$ in the ON state $\left(\psi_{1},\psi_{2}\right)=($ 0,3), and both $\psi_{1}$ and $\psi_{2}$ in the OFF state $\left(\psi_{1},\psi_{2}\right)=($ 0,0) as shown in Figure 3C (right), for d=1 . This situation is more similar to what is seen experimentally in the ESC to DE transition, where activation of core DE TFs genes (GATA6, MIXL1, EOMES, SOX17) induces the inactivation of the core ESC TF genes (POU5F1, SOX2, and NANOG).

### Cell-specific expression of miRNAs

Next, we analyze miRNA expression data to detect miRNAs that are differentially expressed in ESC and DE and might be repressing TFs in the other cell type. PCA of miRNA expression across 177 ENCODE samples (Reese et al., 2023) shows some degree of clustering and separation between samples originating from the three germ layers (endoderm, mesoderm and ectoderm) (Figure 4A; Supplementary Figure S2, Methods). The three human embryonic stem cell samples (hESC) are close to each other in the PCA space, and have an average expression correlation of 0.95 compared to average expression correlation of 0.77 for all 177 pairwise comparisons.

**FIGURE 4:**
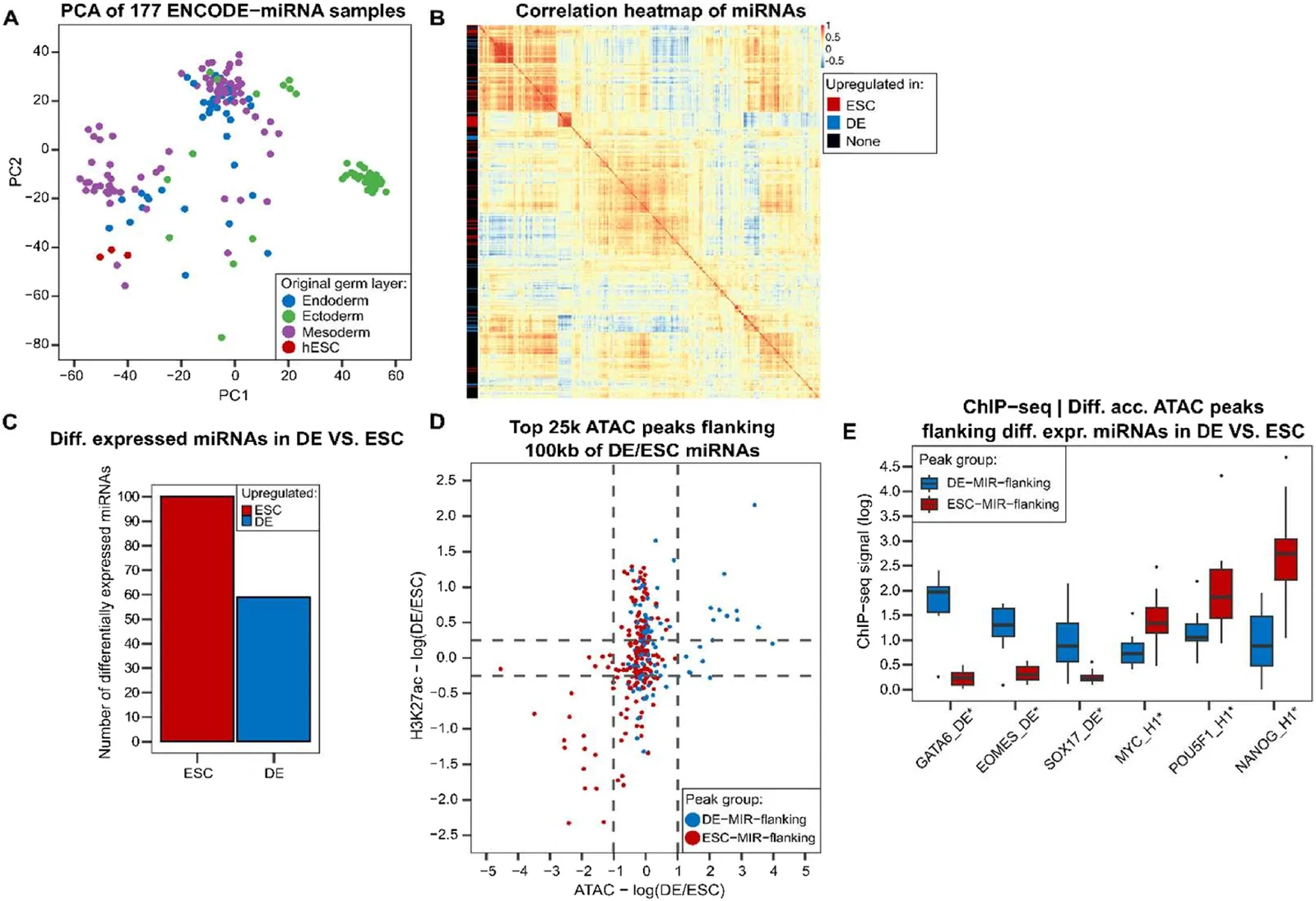
In the transition from ESC to DE, there are multiple miRNAs expressed in a cell-specific manner, flanked by peaks of cell-specific chromatin accessibility consistent with enhancer activity driving the target miRNA expression. **(A)** PCA of miRNA expression in tissues and cell lines identifies clusters of samples. **(B)** Correlation heatmap of 835 miRNAs with detectable expression in multiple samples (Methods). Differentially expressed miRNAs in DE compared to ESC have some similar patterns of expression in ENCODE samples. **(C)** Number of upregulated miRNAs in DE compared to ESC. **(D)** There are differentially active ATAC peaks flanking differentially expressed miRNAs with higher activity in the tissue where the flanking miRNA expression is upregulated. “DE-MIR-flanking” peaks are ATAC peaks having a miRNA upregulated in DE within 100 kb of them. “ESC-MIR-flanking” peaks are defined similarly. **(E)** DE and ESC TFs have higher binding to differentially accessible DE-MIR-flanking and ESC-MIR-flanking peaks, respectively (*t*-test, *p < 0.01). MYC, POU5F, and NANOG ChIP-seq experiments were performed in ESC (H1), while others were done in DE cells. hESC: human embryonic stem cell.

We performed a differential expression analysis for miRNAs in DE compared to the ESC cell line and found 59 miRNAs overexpressed in DE state and 100 miRNAs with higher expression in ESC (Figures 4B, C). Some miRNAs upregulated in either ESC or DE are co-expressed across other tissues and cluster together (Figure 4B), providing evidence that miRNA expression differences are informative and vary across differentiation states and tissues.

### Differential miRNA expression is driven by differential chromatin accessibility and TF binding at putative enhancers

ESC and DE ATAC-seq along with H3K27ac (Li et al., 2019; Luo et al., 2023) shows that peaks flanking differentially expressed miRNAs are consistently more active in the tissue where the flanking miRNAs are overexpressed (Figure 4D, Methods). Core DE and core ESC TFs bind preferentially to the most differentially accessible peaks between DE and ESC that are near a differentially expressed miRNA (Figure 4E). GATA6, EOMES and SOX17 have a significantly higher binding to ATAC peaks flanking miRNAs upregulated in DE (named “DE-MIR-flanking” peaks), whereas MYC, POU5F1 and NANOG have a higher ChIP-seq signal at differentially accessible peaks in ESC flanking ESC miRNAs (“ESC-MIR-flanking” peaks) (*t*-test *p* < 0.01, Figure 4E). In addition, GATA and POU5F1 DNA-binding motif sequences have a significantly higher enrichment in differentially accessible DE-MIR-flanking peaks and ESC-MIR-flanking peaks, respectively (*t*-test *p* < 0.05, Supplementary Figure S3, Methods). Taken together, there are distinct patterns of accessibility and TF binding at peaks near differentially expressed miRNAs in ESC and DE which suggest that they are functioning as enhancers driving the differential expression of these miRNAs in ESC and DE, consistent with our modeling in Figure 3.

### Differentially expressed miRNAs are predicted to target 3′UTRs of core DE and ESC TFs

Our dynamic network modeling (Figures 1, 3) highlights the possibility of miRNAs repressing core ESC and DE TFs during embryonic differentiation. We used TargetScan and mirDIP (McGeary et al., 2019; Tokar et al., 2018) to find the set of candidate miRNAs targeting 3′UTRs of core TFs (Figure 5A, Methods) and discovered a subset of miRNAs predicted to target MYC, SOX2, POU5F1, EOMES and GATA6 (Figure 5B). MIR92B is expressed at a higher level in ESC and is confidently predicted to target EOMES and GATA6 (Figure 5C). MIR145 is upregulated in DE and consistent with the target prediction, MIR145 was shown to repress pluripotency by targeting ESC TFs POU5F1, SOX2 and KLF4 (Xu et al., 2009). MIR892A is upregulated in DE while it has low expression in the ESC state and is predicted to target POU5F1. Another core ESC TF is MYC predicted to be targeted by MIRLET7D (Supplementary Figure S4). In addition, our analysis of differentially expressed miRNAs identified a potential combinatorial regulation of SOX2 by multiple DE miRNAs such as MIR145, MIR369 and MIRLET7F1 (Figure 5D). This potential combinatorial repression is consistent with SOX2 being the core ESC TF having the highest and the fastest downregulation following ESC-DE transition through DE day 3 (Supplementary Figure S1).

**FIGURE 5:**
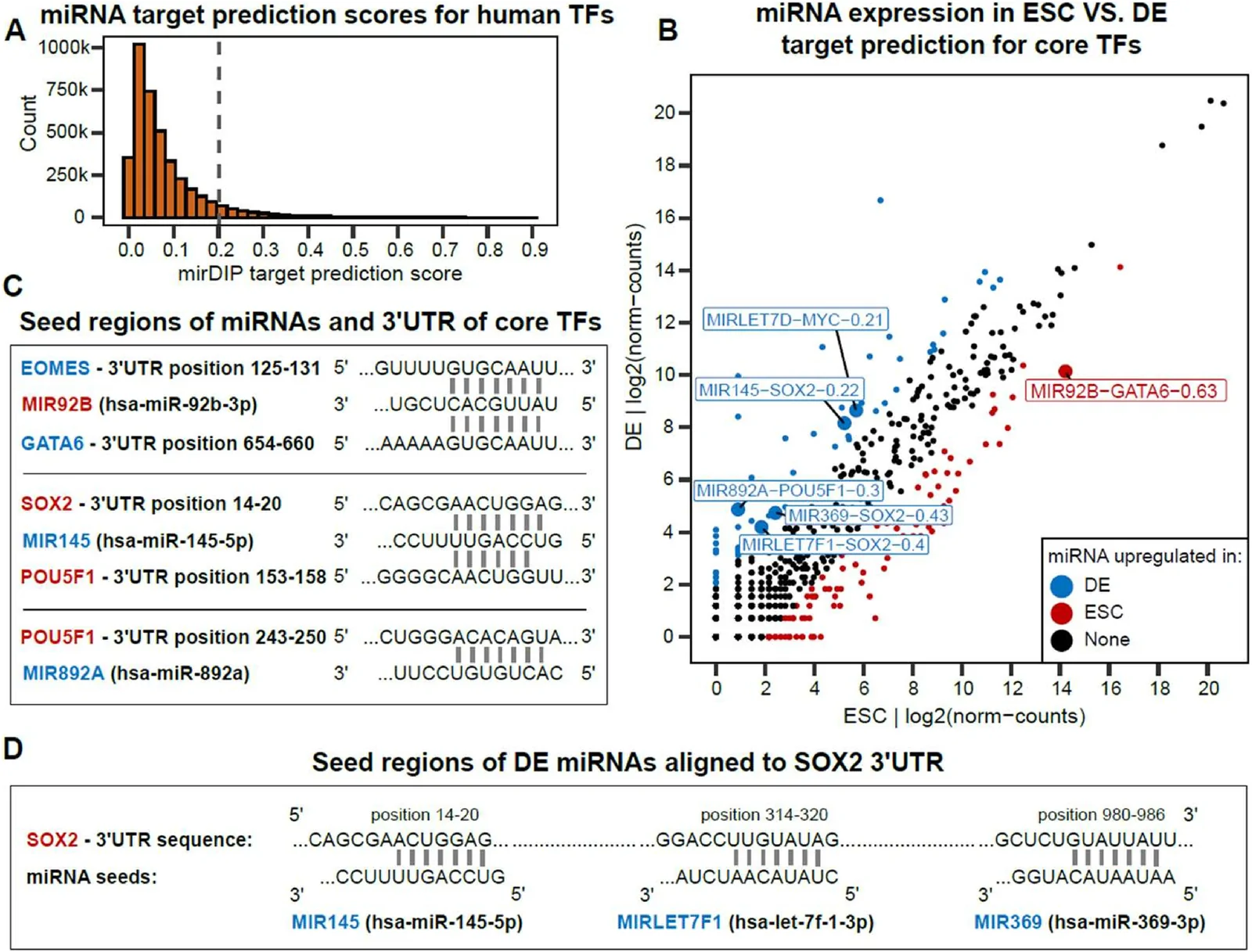
Some differentially expressed miRNAs target 3′ UTRs of core TFs of other cell types, consistent with a negative feedback role in cell-type specification. **(A)** Distribution of mirDIP prediction scores for pairs of [miRNA, TF] where we chose 0.2 < mirDIP_score as the cutoff for miRNA target prediction. **(B)** Differentially expressed miRNAs in DE compared to ESC (|log_2_FC| > 2). Labeled miRNAs are those with a flanking (<100 kb) differentially active ATAC peak which are predicted to target a core DE or ESC TF by mirDIP. Numbers are mirDIP target prediction scores. **(C)** miRNA seed sequences aligned to 3′UTR of DE and ESC TFs. **(D)** The SOX2 3′UTR contains multiple seed target sequences for DE expressed miRNAs, consistent with combinatorial translational repression.

### Chromatin accessibility variations around miRNAs targeting ESC and DE TFs

We identified differentially expressed miRNAs putatively targeting DE or ESC TFs and found differentially active enhancer peaks consistent with their cell-specific expression. MIR92B is predicted to target DE TFs and has a differentially active candidate enhancer (*E1*) flanking it (Figure 6A), where ESC TFs such as POU5F1 and NANOG bind. Conversely, DE TFs such as EOMES, GATA6 and SOX17 bind to the differentially accessible candidate enhancers (*E2* and *E3*) flanking MIR145 and MIR892A (Figures 6B, C). These candidate enhancers are all within loop extrusion model predicted CTCF loops (Xi and Beer, 2021) enclosing the miRNA, providing more support that they are responsible for the miRNA differential expression. Together, these results suggest DE and ESC TFs, through activating *cis*-regulatory elements, upregulate miRNAs, which in turn repress core TFs for the other transition state.

**FIGURE 6:**
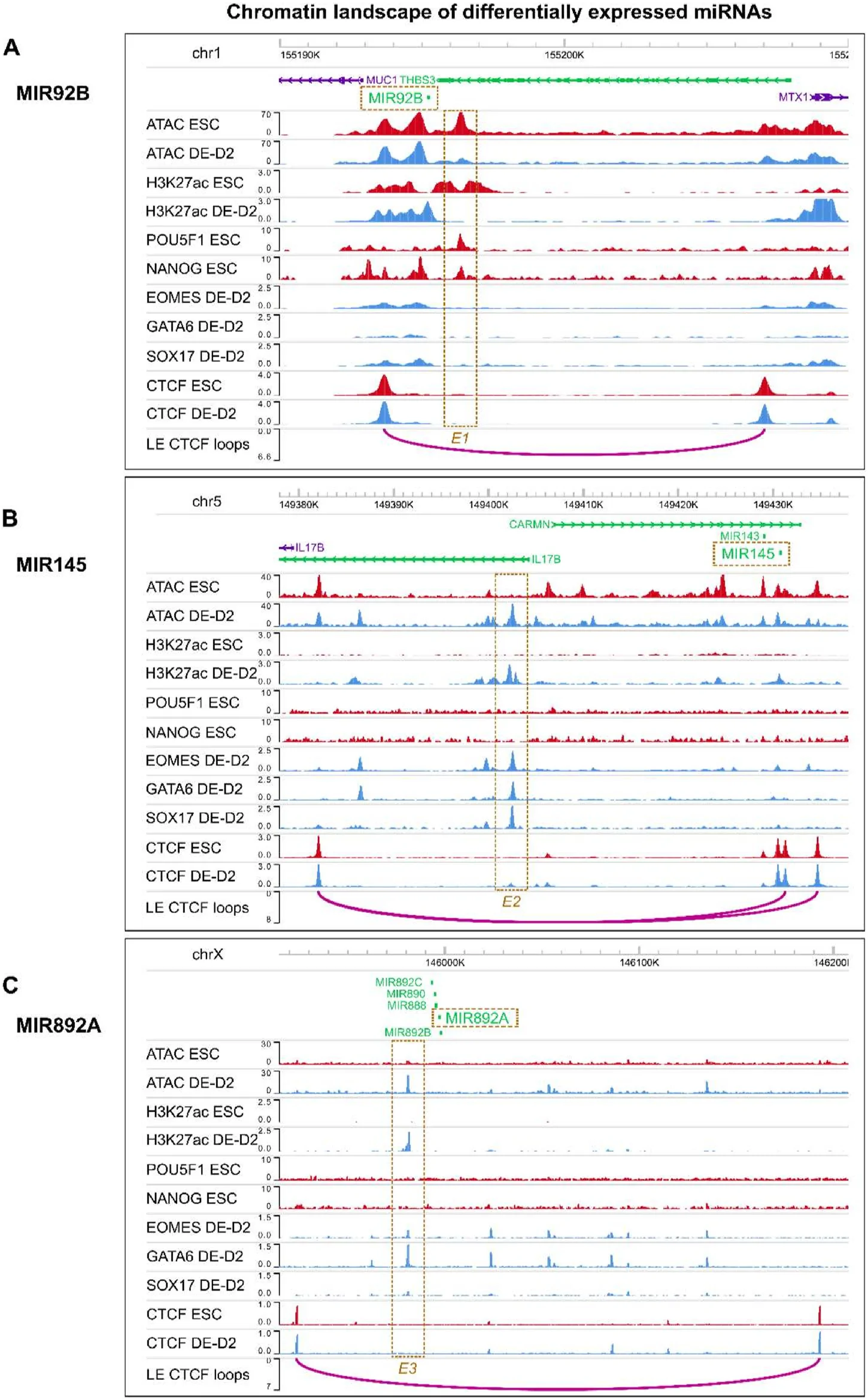
Chromatin accessibility and TF binding at candidate enhancers activating ESC or DE specific miRNAs. Chromatin landscape around **(A)** MIR92B **(B)** MIR145 **(C)** MIR892A. *E1*, *E2* and *E3* are candidate enhancers within the same CTCF loop as the miRNAs. Their accessibility may be regulated through binding of core DE or ESC TFs and these candidate enhancers may control the expression of the nearby miRNAs which will repress TFs of the other transition state.

## Discussion

Cell state transitions are characterized by the activation of lineage-specific core TF genes and the inactivation of at least some of the core TF genes of the precursor cell type. Our modeling shows that a repressive mechanism is required to achieve this inactivation. In our analysis of a very large set of chromatin accessibility datasets, we have found a deficit of repressive sequence signals in enhancers that could provide this shut-off mechanism, relative to activating sequence signals. We see evidence for TFBS for ZEB1/2, SNAI1/2, GLI, ZBTB7A, Nuclear Hormone Receptors, GFI1, REST and others acting as repressors, but in our systematic sequence analysis, there are far fewer repressors detected than activators, suggesting that there are too few repressors to act individually in multiple different cell types to negatively regulate the large set of known activators. On the other hand, it is quite easy to imagine each cell type expressing a small number of miRNAs to repress the TFs in precursor and downstream cell types by targeting the TF’s 3′UTRs. The PRC2 complex or other repressive complexes may play this repressive role by binding promoters, but how these complexes are recruited to different genomic locations in a cell specific manner is unclear. There is also strong autoactivation of core TFs in most tissues, where we have found that core TFs bind their own enhancers. This autoactivation is a key component of our dynamic gene network model, and helps explain our experimental observations of the effect of enhancer perturbation in the ESC-DE transition (Luo et al., 2023). However, the autoactivation also induces hysteresis in the gene regulatory network which makes it difficult to turn off cell-specific core TFs once they are activated. One could argue that the tendency for chromatin to be bound by nucleosomes and revert to a heterochromatic state could provide a generic shut-off mechanism that provides the required negative feedback, but mathematically this would be equivalent to using a lower basal transcription rate, controlled by parameters b and $e_{0}$ in our model, which are already quite small. The repressive effect could also be modeled by requiring stronger cooperativity to overcome the heterochromatic state, by using $c\psi^{4}$ instead of $c\psi^{3}$ , but changing b , $e_{0}$ or the cooperativity exponent does not qualitatively change the stability analysis shown in Figures 3B, C. The point is that once autoactivation is significant enough to overcome any default heterochromatic repression, it remains strong enough to overcome it until another distinct repressive mechanism is induced. In this paper we have provided modeling and analysis of epigenomic datasets in support of miRNAs acting to provide the negative feedback necessary to shut off the core TFs of neighboring cell types and provide stability to cell types and cell state transitions.

Our miRNA model is generic and can apply to many different cell types, but our epigenomic analysis is specific to the ESC to DE transition for which we have ample high-quality datasets. We identified differentially expressed miRNAs in ESC and DE, and differentially active enhancers flanking these miRNAs which are likely responsible for their differential expression and which are bound by the relevant cell-specific core TFs. We found both ESC-expressed miRNAs which are driven by core ESC TF-bound enhancers that provide the translational repression of core DE TF genes through binding sites in their 3′UTRs, and DE-expressed miRNAs which are driven by core DE TF-bound enhancers that carry out the translational repression of core ESC TF genes, again with miRNA binding sites in their 3′UTRs.

More detailed experimental validation of these predictions is required, but if verified, a better understanding of the role of miRNAs in cell-state transitions may allow further improvement in the efficiency of stem cell differentiation for both scientific and therapeutic purposes, and may help better understand the role of miRNAs in cancer and as potential therapeutic targets.

## Methods

### Gkm-SVM modeling

We trained gkm-SVM on ATAC-seq data from ESC and DE (Li et al., 2019), after removing promoter peaks within 2 kb of an annotated TSS, non-cell specific peaks accessible in more than 30% of ENCODE samples, and CTCF binding sites. We removed promoters as they have similar TFBS and are usually active across all cell types (Oh and Beer, 2024). Gkm-SVM uses counts of gapped kmer features under the 300bp sequences centered on the ATAC-seq peaks, as gapped kmers are effective at describing regulatory sequence features (Ghandi et al., 2014b; Amanchy et al., 2011) and do not require previous knowledge of TFBS. Following the default training method in the gkmSVM-R package (Ghandi et al., 2016), the AUROC was evaluated on 5-fold cross validation (CV) and AUROC is the average of 5 CV test sets (0.902 for ESC, 0.949 for DE-D2, and 0.975 for DE-D2 vs. ESC). All cross-fold validation sets produce similar sets of features and have very similar weight vectors. We normalized weight vectors to have zero mean and unit standard deviation for comparison. We extracted TFBS from this weight vector using gkm-PWM (Shigaki, 2024), which produces a lasso weight reflecting the importance of each motif in explaining the full weight vector (W in Figure 2). Other motif metrics Z and I describe the Z-score for kmers mapping to the motif and the error induced by removing the motif from the list.

### Dynamic gene regulatory network modeling

We modified the gene regulatory network model (Luo et al., 2023) to include miRNA repression. The model describes the rate of change of cell-specific TFs, where one cell state’s TF concentrations are described by $\psi_{1}\left(t\right)$ , and the other cell state’s TF concentrations are described by $\psi_{2}\left(t\right)$ . For simplicity, we assume that through autoregulation, each cell state’s TFs turn on or off together, and can thus be represented with one concentration (Luo et al., 2023). The differential equation describes the change in concentration of the TFs, which is driven by the balance between a transcription rate, $T\left(\psi \right)$ , and degradation/export, −rψ , see, for example, (Alon, 2019). For both the TF and miRNA transcription rate we choose the form $T\left(\psi \right)=\frac{e_{1}c\psi^{3}+e_{0}b}{b+c\psi^{3}}$ . This rate depends on the TF activity, ψ , through enhancer activation, and the parameters control the rate at which transcription transitions from low transcription to high transcription as a function of ψ . It is a simple Hill form (Alon, 2019), but can also be understood as a two-state model with a large enhancer activated transcription rate, $e_{1}$ , and a low basal transcription rate, $e_{0}$ . Each cell type’s enhancers are active proportional to $\frac{c\psi^{3}}{b+c\psi^{3}}$ and inactive proportional to $\frac{b}{b+c\psi^{3}}$ . We will assume that miRNAs are similarly activated with enhancers, and repress the other cell type’s core TF activity with a rate d . This rate includes miRNA processing and nuclear transport. The core TFs are degraded and/or exported from the nucleus at a rate r . Together this yields two coupled nonlinear differential equations for $\psi_{1}$ and $\psi_{2}$ as shown in Figure 3A. Transitions from cell type 1 to cell type 2 are modeled (Luo et al., 2023) by adding a time-dependent stimulus, $\delta \left(t\right)$ , which acts at a subset of cell type 2 enhancers to induce activation of $\psi_{2}$ , by increasing the probability that $\psi_{2}$ ’s enhancers are active. The probability that $\psi_{2}$ is in the basal state is b , and the probability that $\psi_{2}$ ’s enhancers are active is $c\psi_{2}^{3}$ without stimulus and $c\psi_{2}^{3}+\delta \left(t\right)$ with the stimulus, with $\delta \left(t\right)=\delta_{0}\left[1+\frac{\mathrm{erf}\left(t-t_{0}\right)}{2\sqrt{2}}\right]/2.$ This stimulus turns on gradually at $t_{0}=3$ . The other parameters are $\left(b,c,e_{0},e_{1},r,d,\delta_{0}\right)=\left(0.5,1,0.1,3,1,1,0\right)$ unless specified otherwise.

### ENCODE miRNA expression data

We downloaded 177 ENCODE miRNA-seq samples from the ENCODE portal (Reese et al., 2023) (Supplementary Table S1). Raw miRNA expression counts were mean-normalized across the 177 samples and log_2_-transformed. 835 miRNAs of the annotated 1877 miRNAs had a log_2_-normalized expression above 4 (∼16 normalized counts) in at least two samples. We used this subset of 835 miRNAs to plot a PCA and heatmap of miRNAs across different tissues. We generated a table assigning a likely germ layer of origin to miRNA-seq tissue and cell line experiments (Supplementary Table S1). PCA and heatmaps were generated in R using ‘prcomp’, ggplot2 and pheatmap. We used ENCSR588ZRK (H1 embryonic stem cells) labeled “ESC” and ENCSR820BMF (endodermal cell originated from H1) labeled “DE” for miRNA expression analysis.

We used a threshold of |log_2_FC| > 2 to call differentially expressed miRNAs between ESC and DE. Ensembl bioMart V.110 and V.111 were used for miRNA gene name conversion and transcription start site annotation (Martin et al., 2023).

#### DE and ESC core TFs and RNA-seq analysis

We used RNA-seq data from GEO GSE213394 (Luo et al., 2023) to define core DE and core ESC TFs. RNA-seq expression values for HUES8 (ESC) and SOX17+ HUES8 (DE) were upper-quartile normalized. Expression values for DE-D3 (DE day 3) and ESC samples were averaged across the three replicated and differentially expressed genes were called using a threshold of |log_2_FC > 1.5|. The list of human TFs was obtained from Lambert et al. (Lambert et al., 2018).

### ATAC and ChIP-seq analysis

ATAC-seq, ChIP-seq (TFs and H3K27ac) fastq and bigwig files were obtained from GEO GSE213394 (Li et al., 2019). They were processed according to the pipeline described by Shigaki et al. (Shigaki et al., 2019). We compared ATAC-seq and ChIP-seq samples in ESC to DE-D2 (day 2), as some TF ChIP-seq data was only available in DE-D2 (and not in DE-D3). We used ATAC-seq and H3K27ac to identify differentially active candidate enhancers regulating differentially expressed miRNAs in DE and ESC. We extended the ATAC peak summit by ± 500bp or 150bp on each side (peak length = 1000bp for H3K27ac and 300bp for ATAC) and calculated the average H3K27ac signal across the extended region, as H3K27ac peaks typically flank the ATAC peak summit. Differentially active regions were called from the set of top 25,000 strongest ATAC peaks in DE and ESC which satisfy the following: i) ATAC |log_2_FC| > 1; ii) H3K27ac |log_2_FC| > 0.25; iii) fall within 100kbp of a differentially expressed miRNA. Peak color indicates whether they are close to a miRNA upregulated in DE (“DE-MIR-flanking” peak) or to a miRNA upregulated in ESC (“ESC-MIR-flanking” peak). Of the 101 DE-MIR-flanking and 183 ESC-MIR-flanking peaks, only seven peaks are shared among the two sets and this subset of peaks has a relatively similar ATAC signal in DE and ESC (not differentially active and not affecting the rest of the analysis).

GATA6, EOMES and SOX17 ChIP-seq samples in DE were obtained from GSE213394, while the bigwig files for MYC (ENCFF145JGY, Snyder Lab), POU5F1 (ENCFF106YHB, Myers Lab) and NANOG (ENCFF512EZC, Myers Lab) ChIP-seq in H1 (ESC) were downloaded from the ENCODE portal (Luo et al., 2020). UCSC bigWigAverageOverBed was used to calculate average ChIP-seq signal over DE-MIR-flanking and ESC-MIR-flanking peaks and the values were log_2_-transformed and *t*-test was used.

### TF motif analysis

For motif enrichment analysis, we used STORM (Schones et al., 2007) and the following PWMs: GATA (MEME “MOTIF GATA1-ext”), GSC (JASPAR “MA0648.1”), SOX (JASPAR “MA0143.4”), EOMES (MEME “MOTIF Eomes”), NANOG (HOMER “motif224”), POU5F1 (JASPAR “MA1115.1”) (Bailey et al., 2015; Fornes et al., 2020; Heinz et al., 2010). *t*-test was used. To remove CTCF binding sites we scored with ScanACE (Karnik and Beer, 2015) with a score threshold of 5.0.

### miRNA target prediction

We used mirDIP to find a set of candidate miRNAs targeting core DE/ESC TFs and selected pairs of miRNA and core TFs whose mirDIP score is greater than 0.20. TargetScan was used to validate mirDIP predictions and align 3′UTRs to miRNA seed regions.

### Genome browser

We used WashU Epigenome Browser (Li et al., 2022) for Figure 6. For better visual clarity in the figure, we used RefGene annotations (genomic coordinates) for MIR145 and MIR92B, while we used GENCODE V39 for MIR892A coordinates.

## Data Availability

Publicly available datasets were analyzed in this study. The datasets can be found here: https://encodeproject.org and https://www.ncbi.nlm.nih.gov/geo/ and the ENCODE (ENCFF) and GEO (GSE) accession numbers can be found in the Methods section.

## Supplementary material

SUPPLEMENTARY FIGURE S1 DE and ESC TFs.

SUPPLEMENTARY FIGURE S2 Correlation heatmap of 177 ENCODE miRNA expression samples.

SUPPLEMENTARY FIGURE S3 DNA binding site (motif) enrichment in loci flanking DE and ESC miRNAs.

SUPPLEMENTARY FIGURE S4 Alignment of seed regions to 3′UTRs of TFs.

SUPPLEMENTARY TABLE S1 ENCODE miRNA expression datasets.
