# Supplementary material for: MicroRNAs provide negative feedback and stability in gene regulatory network models of cell-state transitions
Source: Front Epigenet Epigenom. 2024 Oct 2;2:1473789. doi: 10.3389/freae.2024.1473789 (PMC13004118; doi:10.3389/freae.2024.1473789)
Supplement: Supplementary file 1 [file Image1.pdf]

Fig. S1) DE and ESC TFs

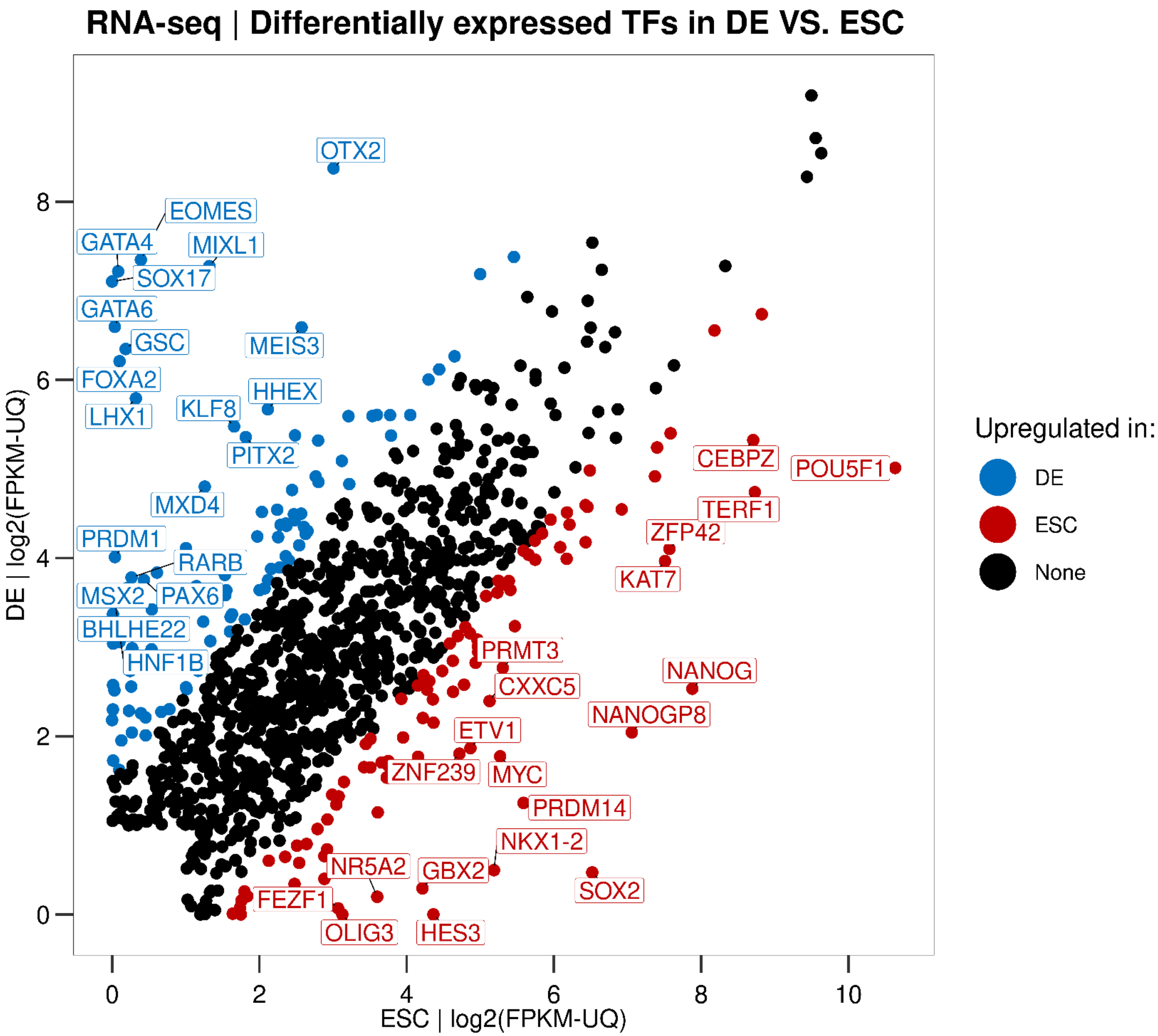


Differentially expressed TFs in ESC (HUES8 cell line) compared to DE-D3 (SOX17+ HUES8, DE day 3). $|\log_2 FC| > 1.5$. Top 20 TFs with the highest fold-changes on each side are labeled.

Fig. S2) Correlation heatmap of 177 ENCODE miRNA expression samples

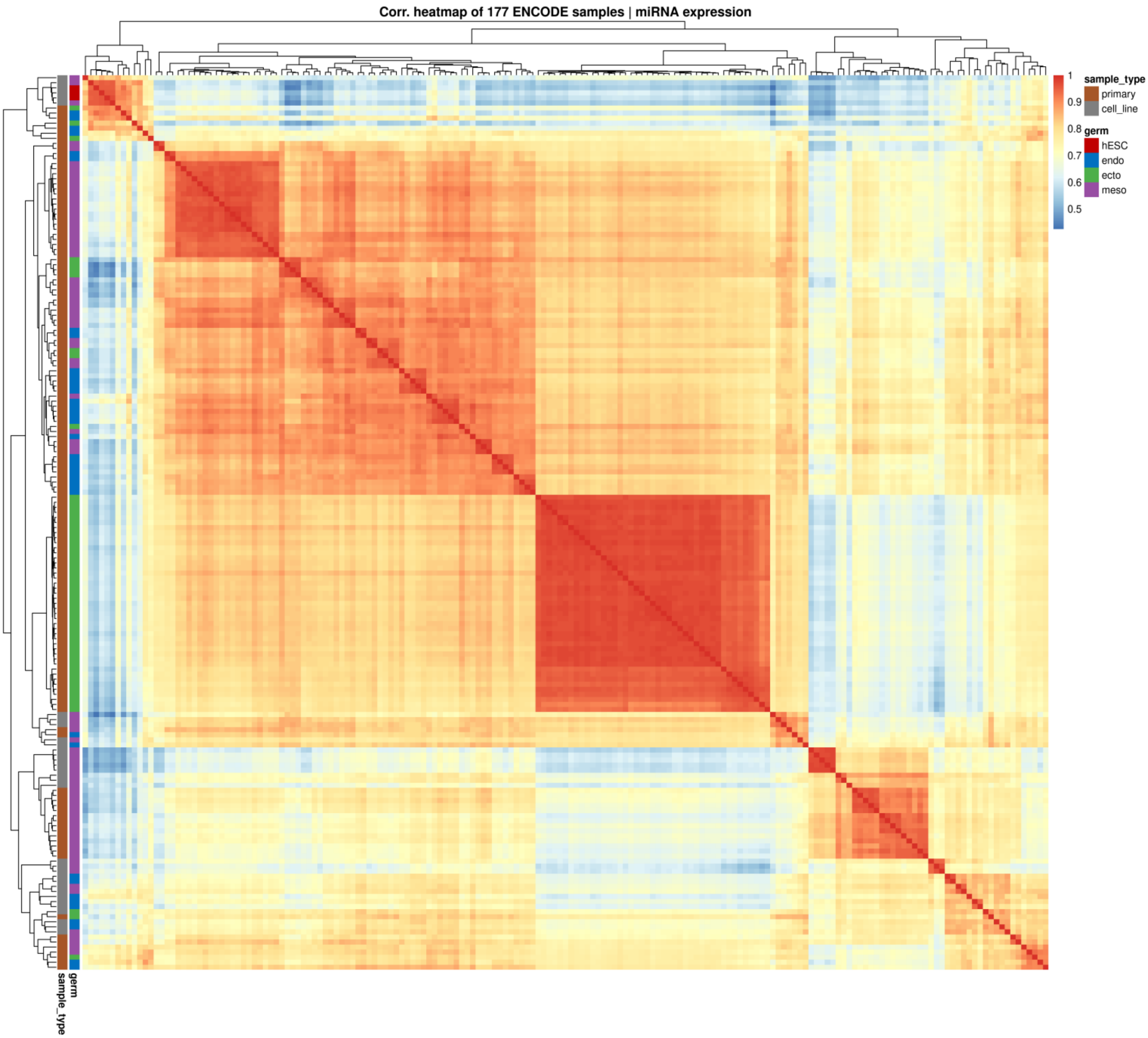


Correlation heatmap of miRNA expression of 177 ENCODE samples, annotated by germ layer of origin and sample type (i.e. primary tissue/cells or cell line).

Fig. S3) DNA binding site (motif) enrichment in loci flanking DE and ESC miRNAs

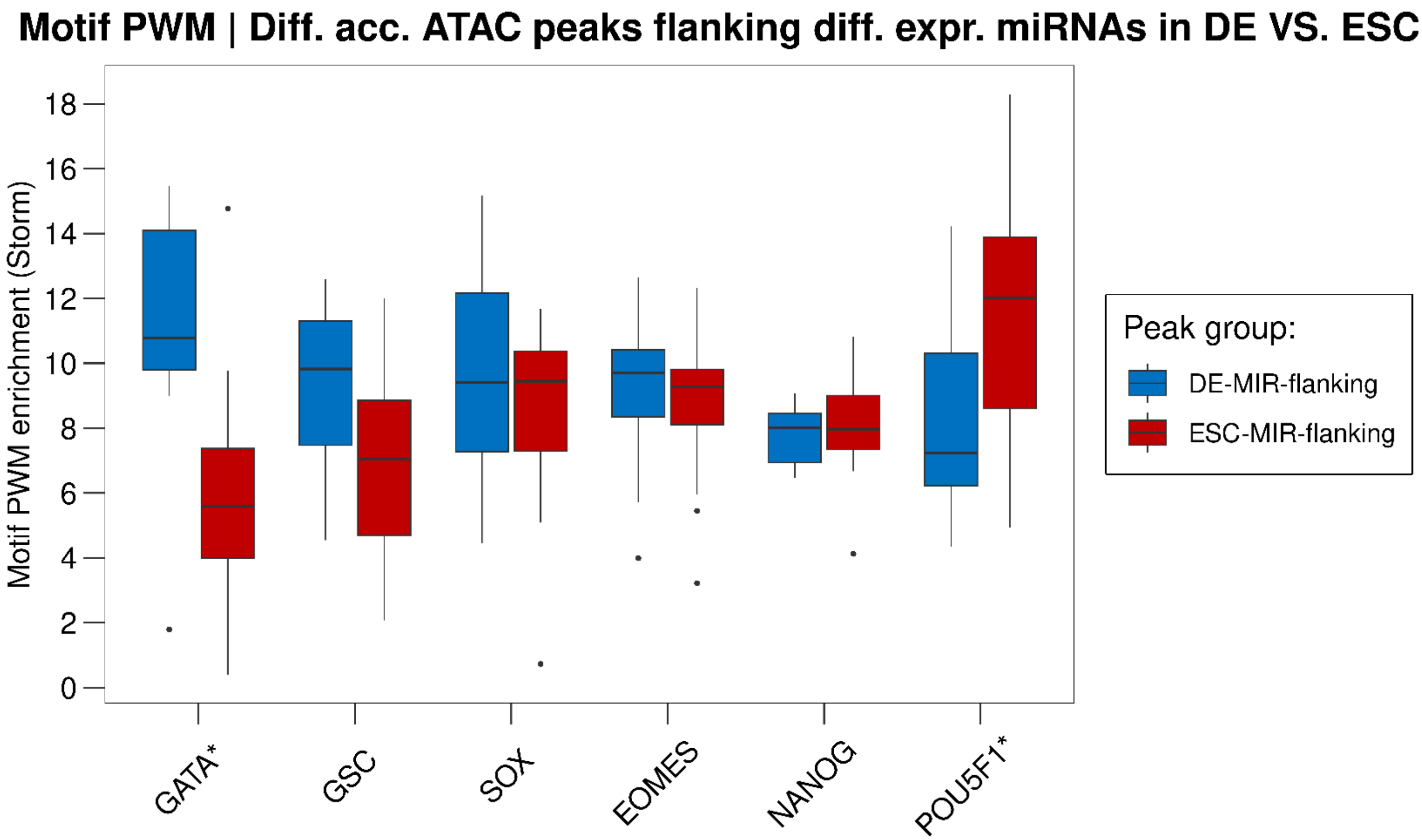


Differentially accessible ATAC peaks flanking DE miRNAs (DE-MIR-flanking peaks) have a significantly higher enrichment for GATA (DE TF, *t*-test $p < 0.05$). Likewise, ESC-MIR-flanking have a significantly higher enrichment of POU5F1 (OCT4, ESC TF).

Position Weight Matrix (PWM) describes binding affinity of a TF. Y-axis is STORM's motif enrichment score.

Fig. S4) Alignment of seed regions to 3’UTRs of TFs

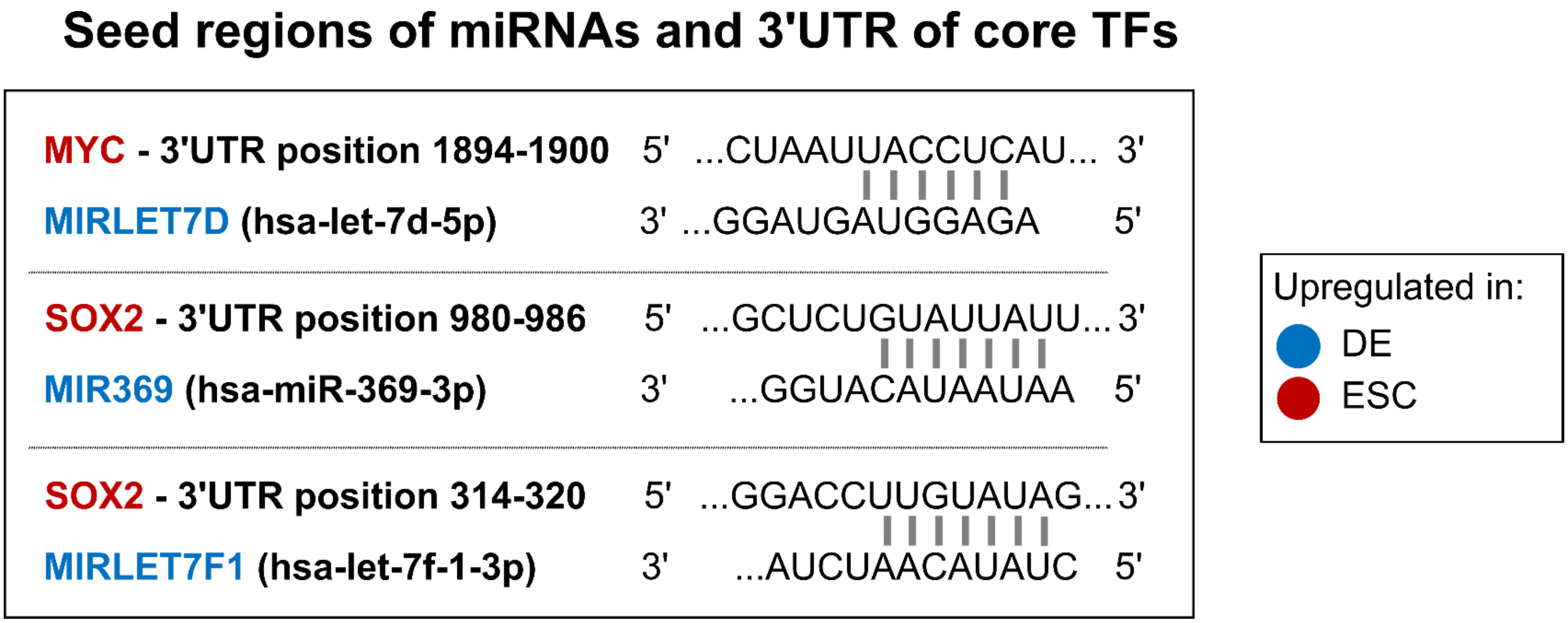


Differentially expressed miRNAs with a flanking differentially active peak. These miRNAs are predicted to target ESC TFs (SOX2 and MYC).
